# An Internet-based self-help intervention for people with HIV and depressive symptoms: study protocol for a randomized controlled trial

**DOI:** 10.1186/s13063-016-1292-6

**Published:** 2016-03-31

**Authors:** Sanne van Luenen, Vivian Kraaij, Philip Spinhoven, Nadia Garnefski

**Affiliations:** Institute of Psychology, Section of Clinical Psychology, Leiden University, Faculty of Social and Behavioral Sciences, P.O. Box 9555, 2300 RB Leiden, The Netherlands

**Keywords:** HIV, Depression, Internet, Self-help, Cognitive behavioral therapy, Coaching, Randomized controlled trial

## Abstract

**Background:**

Many people living with HIV suffer from depressive symptoms. In a previous pilot study, self-help cognitive behavioral therapy (in booklet format) was found to be effective in treating depressive symptoms in people with HIV. We developed an online self-help program in Dutch and English (based on the booklet) for people with HIV and depressive symptoms. Besides the main question regarding the effectiveness of the program aimed at lowering depressive symptoms, sub-questions will focus on the moderators of treatment success (for which patients is the program especially beneficial?) and the mechanisms of change underlying the treatment outcome (which mediators affect the outcome of treatment?). In this paper, the protocol of the study will be described.

**Methods/design:**

The effectiveness of the program will be investigated by comparing the intervention group with a waiting list-control group in a randomized controlled design, by including a pretest and three post-tests. The self-help program contains four main components: activation, relaxation, changing maladaptive cognitions, and goal attainment. Participants with mild to moderate depressive symptoms will work on the program for 6 to 10 weeks, during which a coach will provide motivational support by telephone once a week. Participants in the control condition will receive weekly minimal support from a coach for 8 weeks, and after the second post-test, they can gain access to the self-help program. Depressive symptoms and possible mediators (e.g., activation, cognitive coping, self-efficacy, and goal adjustment) will be assessed by self-report three times during the intervention/waiting period and at the pretest and first post-test.

**Discussion:**

The proposed study aims to evaluate the effectiveness of an online self-help intervention for people with HIV and depressive symptoms. If the intervention is shown to be effective, the program will be implemented. Consequently, many patients with HIV could be reached, and their psychological care may be improved.

**Trial registration:**

Netherlands Trial Register: NTR5407

**Electronic supplementary material:**

The online version of this article (doi:10.1186/s13063-016-1292-6) contains supplementary material, which is available to authorized users.

## Background

Currently, HIV is a chronic disease that requires the use of medication. Along with the physical problems that people living with HIV (PLH) may experience (e.g., linked to the side effects of medication or comorbidities), psychological symptoms are also prevalent [1, 2]. Specifically, depressive symptoms are often reported by PLH. Many PLH struggle, for example, with the daily use of medication and thinking about whom to tell about their HIV [3]. This and more general life stressors may lead to depressive symptoms in PLH. In turn, PLH with depressive symptoms have a higher chance of nonadherence to their medication [4]. This can result in more physical symptoms and illness. Therefore, the treatment of depressive symptoms in PLH is important.

Various treatments are available for PLH with depressive symptoms. Group and individual therapies exist, and many of these contain cognitive behavioral components (see review studies [5–9]). These treatments are effective in decreasing depressive symptoms and improving the quality of life in PLH [2, 5, 7–9]. Furthermore, beneficial effects were also found for medication adherence [6].

These group and individual therapies have some disadvantages. First, patients and therapists should meet in person during office hours for the treatment. Second, the costs of these treatments are high. Third, for HIV-specific programs, the stigma is a significant barrier to service delivery and utilization [10]. A self-help program could be a way to overcome these disadvantages and barriers. The evidence base for low-intensity interventions has been growing in recent years, with high-quality trials supporting the effectiveness of self-help cognitive behavioral therapy (CBT) for depression [11–13].

A low-cost CBT self-help booklet for PLH was developed based on prior research and a needs assessment [14–17]. The self-help booklet contains three main components: relaxation, changing maladaptive cognitions, and goal attainment. The effectiveness of this booklet was tested in a pilot randomized controlled trial (RCT) [18]. The results indicated that this intervention was effective in reducing the depressive symptoms in PLH, compared to waiting list controls. However, in this RCT, 25 % of the participants dropped out during the intervention phase. Elements should therefore be added to retain more PLH in the program and to increase the effectiveness. In addition, several participants indicated they were worried that having the booklet in their home would disclose their HIV-positive status. They would have valued a less stigmatizing mode.

Therefore, the self-help booklet was converted into an Internet version. The online program contains eight lessons, which can be completed in 6 to 10 weeks. An online program has a lower stigma, is easily accessible, can reach more people, and has lower long-term costs. Previous studies showed that online self-help therapy for depression in people with chronic illness was effective [19]. In addition to being converted from a booklet into an Internet version, the program was expanded in three ways in the proposed study:

1. An activation component was added, in which participants will be stimulated to perform (small) activities (e.g., wake up on time). Activation is often used in CBT for depression as a first step [20].
2. Minimal coaching with motivational interviewing will be offered. A meta-analysis [12] showed a greater effectiveness of the Internet therapy when coaching was included, also when given in a minimal format. In addition, our recent studies including minimal coaching showed attrition rates between 10 and 15 % [21].
3. Finally, in the current study, the online intervention will be available in Dutch and English to reach more PLH in the Netherlands (and possibly in other countries in the future).

In 2014, the development of the online program was finalized, and we asked a focus group (four volunteers from the Dutch HIV Association) to evaluate the program. Thereafter, we adapted the program, and we conducted a pilot study with 20 PLH with depressive symptoms (14 males; mean age = 48.65 years, *SD* = 10.86) to test the feasibility of the program. Participants were approached via advertisements on the website and in the magazine of the Dutch HIV Association and were representative of the Dutch HIV population. The results of the pilot study showed that the program was effective in this small group; depressive symptoms decreased from pre-test to post-test. Furthermore, participants thought the program was easy to use and helpful to them. These results encouraged us to execute a large study on the effectiveness of the online self-help program.

The aim of the present paper is to describe the study protocol. The proposed study is a randomized controlled trial; the online self-help program will be compared with a waiting list control condition. The study will include a pretest and three post-tests for the intervention condition (directly after the intervention and 3 and 6 months afterward) and a pretest and two post-tests (directly after the 8-week waiting period and 3 months afterward) for the control condition. After the second post-test (5 months after the pretest), the participants in the control group can also begin the program. Concluding, we will investigate the effectiveness of the online self-help program in decreasing depressive symptoms in the short and long term. In addition, the moderators (for which patients is the program especially beneficial?) and mediators (which mechanisms affect the outcome of treatment?) will be assessed.

## Methods/design

### Design

The study is a randomized controlled trial; participants will be randomly allocated to the intervention or the control group. Stratified randomization by treatment center and sex will be conducted. Stratification by sex is conducted, as there will be more male participants in the study, and the allocation of approximately the same number of females to the intervention group as to the control group is preferred. In addition, stratification by treatment center is being conducted because the number of (included) patients will differ among the treatment centers. Approximately equal allocation to each of the groups also in the small treatment centers, is important. This will be realized by creating random number tables for each treatment center, divided by sex. Randomization will take place in blocks of 12 participants for each treatment center. Males and females will represent separate blocks, with six males and six females for each treatment center, of which half will be randomly allocated to the intervention group, and half will be allocated to the control group. Participants, coaches, and researchers cannot be blind for allocation to conditions. Participants will be allocated to one of the conditions after the pretest. The random number tables will be created by an independent researcher, and the allocation sequence will be concealed from the main researcher, who enrolls and informs participants.

**Fig. 1:**
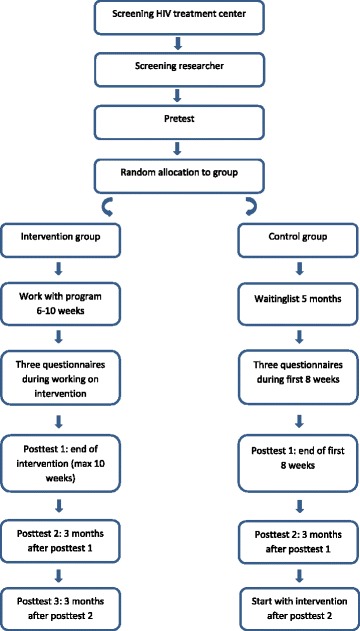
Flow chart of study design

The design consists of multiple measurements, one pretest, three post-tests (two in the control group), and three assessments during the intervention/waiting period. Figure 1 shows a flow chart of the study design, and the SPIRIT Checklist is presented in Additional file 1. The study is approved by the medical ethics committee of the Leiden University Medical Center (LUMC; nr. P14.091). Online informed consent will be obtained from all participants.

### Participants

At least 200 participants who are PLH with mild to moderate depressive symptoms will be included. Participants will be approached in 23 of the 27 HIV treatment centers throughout the Netherlands during their regular visits to the HIV nursing consultants and doctors. To be eligible to participate in the study, a participant must meet all of the following criteria: being HIV positive, exhibiting mild to moderate depressive symptoms (defined as Patient Health Questionnaire 9 (PHQ-9) [22] score > 4 and < 20), age 18 and older, having sufficient knowledge of the Dutch or English language, able to access the Internet, having an e-mail address, and available for the next 8 weeks to work on the intervention.

An eligible participant who meets any of the following criteria will be excluded from participation in the study: being in the first half year following HIV diagnosis, having severe cognitive impairments (e.g., forgetfulness), exhibiting severe depressive symptoms (defined as PHQ-9 score of 20 or higher), experiencing suicidal ideation (indicated by a score > 1 on the suicide item of the PHQ-9), showing an absence of depressive symptoms (indicated by a PHQ-9 score of 4 or lower), undergoing treatment presently by a psychologist or psychiatrist, or on antidepressants for less than 3 months or has experienced a change of type or dose of antidepressants in the past 3 months. In case of severe depressive symptoms (using the set criterion) and/or suicide ideation, patients will be referred to their general practitioner or the HIV treatment center.

### Sample size

A power analysis was performed (with the program Power Analysis and Sample Size Software: PASS; [23]) to determine the sample size needed to assess the long-term effect of the intervention on the primary outcome depressive symptoms. Based on a medium effect size of 0.50, which proved to be realistic according to the previous pilot RCT, and assuming an alpha of .05 and a power of .80, both groups should consist of 64 participants who complete the four measurements. This sample size is also large enough to detect moderate pre-defined two-way cross-product interactions, such as the interactions of group by sex. Taking into account a dropout rate of 15 % during working on the self-help program (based on our recent studies, which included coaching [21]), at least 150 patients should be allocated to the two groups at baseline (*n* = 75 in each group). We will aim for at least 200 participants because dropout will also occur during the follow-up.

### Procedure

HIV nursing consultants and doctors in the treatment centers will screen as many PLH as possible for the level of depression at regular check-ups by means of the PHQ-2 [24]. In some of the treatment centers, the Hospital Anxiety and Depression Scale (HADS [25]) will be used instead of the PHQ-2 because this scale was already in use in these centers. HIV treatment centers will receive information about the study and a guideline for the screening. A form will be used for the screening, and the forms will be sent to the researchers. Patients will be screened over approximately 6 months; most patients visit the HIV treatment center once every 6 months. If the PHQ-2 score is > 0, patients may be referred to the researchers. Patients will receive written information about the study and permission will be asked to provide the researchers with their e-mail addresses and telephone numbers, so the researchers may contact the patients. The researchers will provide additional information to the patients and screen them more extensively with the PHQ-9. If the depression score is mild (PHQ-9 > 4) to moderate (PHQ-9 < 20), patients are eligible for the study and will be invited to participate. After giving online informed consent, participants will be asked for permission to inform their general practitioner and the HIV treatment center about participation in the study. Thereafter, all participants will complete the pretest via online questionnaires (T0). Next, participants will be randomly allocated to either the intervention or the control condition. At the start of the intervention, the personal coach will contact the participant by telephone in order to check and improve motivation by using the technique of motivational interviewing.

Subsequently, participants in the intervention group will follow the self-help program for 6 to 10 weeks and receive support from a coach (see study conditions). They will be asked to fill out online questionnaires three times during the intervention, after they complete the program (T1, a maximum of 10 weeks after T0), 3 months later (T2), and again 3 months later (T3). Participants who are allocated to the control condition will receive minimal support for 8 weeks (for details see study conditions). They will also be asked to fill out the questionnaires, including the T2. After the T2 (which is completed approximately 5 months after T0), the participants will also receive the online intervention. Coaches will monitor the well-being of participants in both groups (see ethical precautions). We will raffle off gift cards and iPods among the participants who completed all questionnaires during the study.

### Study conditions

#### Online self-help intervention

The self-help intervention is grounded in the theories of self-regulation and stress-coping, incorporating techniques of CBT and stress-management. The content of the program reflects four main components covered in eight lessons: activation, relaxation, changing maladaptive cognitions, and goal attainment. The program contains a combination of psycho-education, exercises, and assignments, all strengthened by motivation and support to increase the effectiveness and to minimize the attrition over time.

The intervention is offered through a secured website that contains the complete work program. Participants work on the intervention 1-2 hours a week for a period of 6-10 weeks. The program starts with an introduction, and participants are asked to think of a small activity to perform (e.g., taking a short walk; lesson 1). They are stimulated to perform this activity in the following weeks and to expand this to other activities. Furthermore, participants will be instructed to do relaxation exercises and to continue these exercises in the coming weeks (lesson 2). Thereafter, participants learn to identify and change irrational cognitions (by challenging negative thoughts) and to evoke a strong and positive feeling when they experience negative feelings (lessons 3-5). Furthermore, participants are guided to formulate a new, realistic, concrete goal (such as quit smoking) and work on the stepwise attainment of this goal (lessons 6-7). Finally, the participant will work on a concluding and summarizing lesson (lesson 8). All participants will receive support from a coach during the intervention (see below).

#### Waiting list control condition

Participants randomized to the control group will be placed on a waiting list and will receive the intervention after T2. They will receive an initial phone call to explain the procedures (to minimize attrition). Participants will receive minimal support from a coach (see below).

#### Support from a coach

Participants in the intervention group will be allocated to a coach, who will provide motivation and support throughout the program. During the first telephone contact, the coach will introduce the program, motivate the participant, and address technical aspects. Once a week, a prescheduled telephone call of approximately 15 minutes will be arranged between the participant and the allocated coach. Coaching will be provided through queries about the participant’s progress, motivational remarks, support in case problems are encountered, and encouragement to continue the program. Coaching will not include formal psychotherapy and will be provided until the participant completes the program or for a maximum of 10 weeks. If the participant is not finished with the program after 10 weeks, the coaching will end, but the participant can continue the program.

Participants in the control group will also be allocated to a coach, who will provide minimal support over 8 weeks. Participants will have a pre-scheduled weekly telephone call of approximately 5 minutes with the coach. The coach will address the well-being of the participant. Coaches will try to prevent participant dropout and monitor their depressive symptoms. When depressive symptoms or suicidal thoughts increase, the coach will act appropriately (see ethical precautions).

A protocol for coaching will be used, with guidelines available for the provision of support. Coaches will receive a short training to inform them about the study and the coaching procedures. After each telephone call, coaches will note the elements that were used during the call (e.g., motivation, support) to monitor treatment integrity. Coaches will be Master students in clinical psychology or individuals with a Master’s degree in the field of psychology. The coaches will have completed clinical courses during their Master’s degree program in which they learned communication skills, interview techniques, and treatment strategies. In addition, coaches will be selected in a personal interview on the basis of adequate communication skills for the support and motivation of participants. Coaches will be supervised by the researchers and a psychotherapist. Once every 2 weeks (every week during the first month of the study), the coaches and the main researcher will hold an intervision meeting. Coaches can discuss difficulties and questions with each other and the researcher. If a problem arises between intervision sessions, the coach may contact the researcher. The researcher and the coach can consult a psychotherapist if necessary.

### Ethical precautions

Coaches will monitor the well-being of participants in both the intervention and control groups. Each week, they will ask how the participant is doing. When depressive symptoms worsen or when suicidal thoughts increase, the coach will discuss this with the participant. The coach can refer the participant to the general practitioner or the HIV treatment center, if necessary. The coach can discuss this with the researcher and the psychotherapist. When a participant is referred for more intensive treatment from a psychologist or psychiatrist, the participant may continue with the study. As this may influence the outcomes, the referral will be added as a covariate in the analysis.

Participants will complete questionnaires to assess depressive symptoms during the course of the study. When depressive symptoms of a participant increase, the researcher will notify the coach about this. The coach will discuss this increase of symptoms with the participant and will act appropriately. These guidelines are also described in the protocol for coaches.

### Assessments

All assessments are self-reports and will be conducted online (except for the PHQ-2, which will be on paper for screening by the HIV nursing consultants and doctors and the PHQ-9, which will be used by the researchers for screening by telephone). Table 1 depicts all the assessment instruments that will be used in each stage of the study. The primary outcome is depression severity, as measured by the PHQ-9 [22] and the Center for Epidemiologic Studies Depression Scale (CES-D [26]). Secondary outcomes are physical tension, activation (Behavioral Activation for Depression Scale (BADS) [27]), cognitive reappraisal (Emotion Regulation Questionnaire (ERQ) [28]), cognitive coping (Cognitive Emotion Regulation Questionnaire (CERQ) [29]), depressive thoughts (Crandell Cognitions Inventory (CCI) [30]), behavioral coping (Behavioral Emotion Regulation Questionnaire (BERQ) Kraaij & Garnefski, unpublished questionnaire), coping self-efficacy (Kraaij & Garnefski, unpublished questionnaire), goal adjustment (Goal Disengagement and Reengagement Scale [31]), personal growth (Garnefski & Kraaij, unpublished questionnaire), symptoms of anxiety (Generalized Anxiety Disorder 7 (GAD-7) [32]), negative life events (Life Events Scale, [33]), motivation to start with the intervention, compliance, dropout and reasons for dropout, medical data, and user satisfaction.

**Table 1:** Overview of assessments during the study

Assessment	Screening HIV treatment centers *(+/- 5 minutes)*	Screening researchers *(+/- 10 minutes)*	T0: Pretest *(+/- 25 minutes)*	Three times during intervention/waiting period *(+/- 10 minutes)*	T1: post intervention/waiting period: 6-10 weeks after T0 *(+/- 20 minutes)*	T2: 3-month follow-up *(+/- 20 minutes)*	T3: 6-month follow-up^1^ *(+/- 20 minutes)*
PHQ-2 or HADS	X	-	-	-	-	-	-
PHQ-9	-	X	X	-	X	X	X
CES-D	-	-	X	-	X	X	X
Demographics and other information	-	-	X	-	X	X	X
Physical tension questionnaire	-	-	X	X	X	X	X
BADS	-	-	X	X	X	X	X
ERQ	-	-	X	X	X	X	X
CERQ	-	-	X	X	X	X	X
CCI	-	-	X	-	X	X	X
Self-efficacy questionnaire	-	-	X	X	X	X	X
Goal disengagement and reengagement scale	-	-	X	X	X	X	X
BERQ	-	-	X	-	X	X	X
Personal growth questionnaire	-	-	X	-	X	X	X
GAD-7	-	-	X	-	X	X	X
PHQ-4	-	-	-	X	-	-	-
Life Events Scale	-	-	X	-	X	X	X
Motivation	-	-	X	-	-	-	-
Compliance	-	-	-	X	X	X^1^	X
Dropout	-	-	-	X	X	X	X
Medical data	-	-	X	-	-	-	X
User satisfaction questionnaire	-	-	-	-	X	-	-

*PHQ-2* Patient Health Questionnaire 2, *HADS* Hospital Anxiety and Depression Scale, *PHQ-9* Patient Health Questionnaire 9, *CES-D* Center of Epidemiologic Studies Depression Scale, *BADS* Behavioral Activation for Depression Scale, *ERQ* Emotion Regulation Questionnaire, *CERQ* Cognitive Emotion Regulation Questionnaire, *CCI* Crandell Cognitions Inventory, *BERQ* Behavioral Emotion Regulation Questionnaire, *GAD-7* Generalized Anxiety Disorder 7, *PHQ-4* Patient Health Questionnaire 4

^1^Not sent to participants in the control group

As potential moderators of treatment outcome demographic variables (sex, age, education, and nationality), clinical and psychological characteristics (severity of depressive symptoms at baseline, anxiety symptoms at baseline, coping self-efficacy, physical health, HIV status, motivation, and alcohol and drug use) will be tested at first. Previous research indicated that some of these moderators (e.g., sex and baseline depression severity) might be important in online CBT for depression [34, 35]. In addition, other possible moderators will be investigated exploratory.

The mediator variables and the dependent variable will be measured three times during the intervention/waiting period, at the pretest, and at the first post-test. The following mediator variables will be assessed in the study: activation (two items of the BADS), physical tension (two items), cognitive reappraisal (two items of the ERQ), cognitive coping (12 items of the CERQ), goal adjustment (two items of the Goal Disengagement and Reengagement Scale), symptoms of anxiety (PHQ-4 [36]), and coping self-efficacy (two items). The dependent variable in the mediational analysis is the depressive symptoms (PHQ-4). The questions (regarding, for example, symptoms and cognitions) that will be asked during the intervention/waiting period will refer to the last week. The assessments will be administered three times during the intervention/waiting period because, at these moments, we expect change in the mediating variables. The assessments are linked to the lessons of the self-help program for participants in the intervention group. At the first measurement, we expect a change in the behavioral activation; at the second measurement, we expect a change in the physical tension; at the third measurement, we expect a change in the cognitive reappraisal and cognitive coping; and at the post-test, we expect a change in the goal adjustment. Coping self-efficacy and anxiety are expected to change over the entire course of the treatment.

#### Patient Health Questionnaire 9 (PHQ-9)

Depressive symptoms will be measured with the PHQ-9 at the pre- and post-tests [22]. The questionnaire consists of nine items and includes the DSM-V criteria for a major depressive disorder. The items are rated on a four-point scale ranging from 0 “not at all” to 3 “nearly every day,” e.g., “Over the last two weeks, how often have you been bothered by: little interest or pleasure in doing things?” Scores range from 0 to 27, and cutpoints of 5, 10, 15, and 20 represent mild, moderate, moderately severe, and severe levels of depressive symptoms. The PHQ-9 is sensitive to change, has good sensitivity and specificity for detecting depressive disorders, and has adequate psychometric properties [37]. Furthermore, the questionnaire is often used with PLH [38]. This questionnaire will be used to screen participants on depressive symptoms and to measure change in depressive symptoms from baseline to the post-tests.

#### Center of Epidemiologic Studies Depression Scale (CES-D)

The CES-D [26] will be used to measure depressive symptoms during the past week. The questionnaire consists of 20 items, such as “During the past week: I felt sad.” Items are rated on a four-point scale ranging from 0 “rarely or none of the time (less than 1 day)” to 3 “most or all of the time (5-7 days).” Total scores can range from 0 to 60, and the cut-off score for clinical depression is 16. The internal consistency, reliability, and convergent validity of the CES-D are adequate [26, 39], and sensitivity and specificity are high [40]. Furthermore, the questionnaire is often used with PLH [41]. We will use this questionnaire to measure the change in depressive symptoms from the pretest to the post-tests.

#### Demographic variables and other information

A self-designed questionnaire will be used to ask participants about sex, age, educational level, nationality, marital status, sexual orientation, social support, disclosure of HIV positive status, alcohol and drug use, previous depressive and anxiety episodes, treatment of psychological symptoms, and physical health. This information will be used to describe the demographic and clinical characteristics of the sample. In the follow-up assessments, questions will be asked about the use of medication for psychological problems and treatment received from a psychologist or a psychiatrist since the start of the study. The use of medication and treatment received will be added as covariates in the analysis.

Furthermore, participants will complete a self-designed questionnaire about information regarding the HIV infection. Questions include time since the HIV diagnosis, how the infection occurred, symptoms of the infection, cluster of differentiation 4 (CD4) cell count, viral load, use of highly active antiretroviral therapy (HAART), medication adherence, and side effects of the medication. This information will be used to describe the HIV status and use of HAART by the sample.

#### Physical tension questionnaire

Physical tension will be measured with a self-designed 10-item questionnaire. Questions include difficulty to relax, ways to relax, and symptoms of physical tension (e.g., trembling hands, tense neck and shoulder muscles, and increased heart rate). Items can be answered on a three-point scale (no, sometimes, and yes). Higher scores reflect less physical tension.

#### Behavioral Activation for Depression Scale (BADS)

The subscale activation of the BADS [27] will be used to measure activation during the past week. The subscale consists of seven items that will be measured on a seven-point scale ranging from 0 “not at all” to 6 “completely,” e.g., “I am content with the amount and types of things I did.” Higher scores reflect greater levels of activation. The psychometric properties of the Dutch BADS are adequate [42].

#### Emotion Regulation Questionnaire (ERQ)

The reappraisal subscale of the ERQ [28] will be used to measure the cognitive reappraisal. The six items are rated on a scale ranging from 1 “strongly disagree” to 7 “strongly agree,” e.g., “When I want to feel less negative emotion, I change the way I’m thinking about the situation.” Higher scores indicate that cognitive reappraisal is often used to regulate emotions. The convergent and discriminant validity of the ERQ are adequate [28].

#### Cognitive Emotion Regulation Questionnaire (CERQ)

The subscales rumination, catastrophizing, positive refocusing, refocus on planning, positive reappraisal, and putting into perspective of the CERQ [29] will be used to measure the use of these cognitive coping strategies when thinking about having HIV. The subscales consist of four items each, which will be rated on a five-point scale ranging from 1 “(almost) never,” to 5 “(almost) always.” Higher scores on a subscale indicate that this cognitive coping strategy is often used when thinking about having HIV. Examples of items include “I keep thinking about how terrible it is that I have HIV” (catastrophizing) and “I think about pleasant experiences” (positive refocusing). The psychometric properties of the CERQ are adequate [43].

#### Crandell Cognitions Inventory (CCI)

The hopelessness subscale of the CCI [30] will be used to measure black and white depressive thoughts. The subscale consists of seven items that are rated on a five-point scale ranging from 1 “(almost) never” to 5 “(almost) always,” e.g., “I’ll never feel good again.” Higher scores indicate a higher frequency of black and white depressive thinking. The reliability and validity of the CCI are good [30, 44].

#### Coping self-efficacy questionnaire

Self-efficacy to cope with having HIV will be measured with a self-designed eight-item questionnaire (Kraaij & Garnefski, unpublished questionnaire). The questionnaire is based on the Generalized Self-Efficacy Scale, which has a good reliability and validity [45]. Items will be measured on a five-point scale ranging from 1 “totally disagree” to 5 “totally agree,” e.g., “I have the necessary skills to deal with having HIV.” High scores reflect more self-efficacy to cope with having HIV.

#### Goal Disengagement and Reengagement Scale

Goal adjustment will be measured with the Goal Disengagement and Reengagement Scale [31]. We slightly adapted the scale so that questions will be asked regarding the disengagement and reengagement of goals that one has to stop pursuing because of having HIV. The scale consists of four items that measure goal disengagement (e.g., “If I have to stop pursuing an important goal in my life because I have HIV, it’s easy for me to reduce my effort toward the goal”) and six items that measure goal reengagement (e.g., “If I have to stop pursuing an important goal in my life because I have HIV, I start working on other new goals”). Items are measured on a five-point scale ranging from 1 “totally disagree” to 5 “totally agree.” Higher scores indicate more goal disengagement and reengagement. A question about new goals that have already been found will be added: “I have already found new goals.” This last question will be handled separately, with higher scores indicating that more new goals were found.

#### Behavioral Emotion Regulation Questionnaire (BERQ)

The subscales seeking distraction, actively approaching, seeking social support, and withdrawal from the BERQ (Kraaij & Garnefski, unpublished questionnaire) will be used to measure the use of these behavioral strategies to cope with having HIV. Each subscale consists of four items that will be measured on a five-point scale ranging from 1 “never/hardly ever” to 5 “(nearly) always,” e.g., “I do other things to distract myself.” Higher scores on a subscale indicate that the strategy is often used to cope with having HIV. A previous study (Kraaij & Garnefski, in preparation) has shown a good reliability of this questionnaire.

#### Personal growth questionnaire

Personal growth due to having HIV will be measured with a self-designed five-item questionnaire (Garnefski & Kraaij, unpublished questionnaire). Items will be rated on a five-point scale ranging from 1 “not at all” to 5 “certainly,” e.g., “Because of having HIV I appreciate life more.” High scores indicate personal growth due to having HIV. In a previous study, the reliability of this questionnaire was adequate [15].

#### Generalized Anxiety Disorder 7 (GAD-7)

The severity of symptoms of anxiety will be investigated with the GAD-7 [32]. The questionnaire consists of seven items that are rated on a four-point scale ranging from 0 “not at all” to 3 “nearly every day,” e.g., “Over the last 2 weeks, how often have you been bothered by: feeling nervous, anxious or on edge.” Higher scores indicate more anxiety symptoms. The psychometric properties of the GAD-7 are adequate, and the scale also may be used as a screener for panic, social anxiety, and post-traumatic stress disorder [37].

#### Patient Health Questionnaire 2 and Patient Health Questionnaire 4 (PHQ-2 and PHQ-4)

The PHQ-4 [36] will be used to assess depressive and anxiety symptoms during the intervention or waiting period (for the mediational analysis). It consists of the first two questions of the PHQ-9 and the first two questions of the GAD-7. Items are rated on a four-point scale ranging from 0 “not at all” to 3 “nearly every day.” Higher scores indicate more pathology. The internal reliability, construct validity, and factorial validity are adequate [36]. The PHQ-2 [24] consists of the first two questions of the PHQ-9 and will be used by the HIV treatment centers to screen for depressive symptoms.

#### Hospital Anxiety and Depression Scale (HADS)

The HADS [25] will be used by some of the HIV treatment centers to screen for depressive symptoms. The HADS consists of an anxiety and a depression subscale, and each subscale consists of seven questions, e.g., “I still enjoy the things I used to enjoy.” Items are rated on a four-point scale, and the response categories are different for each question. Higher scores indicate more anxiety and depressive symptoms. The HADS is widely used and is a reliable and valid questionnaire to screen for anxiety and depressive symptoms [25, 46].

#### Life Events Scale

Life events will be assessed with the Life Events Scale [33]. The scale consists of 17 negative life events (e.g., divorce of parents), and participants indicate if they have not experienced the life event, experienced it longer than 1 year ago, or experienced it last year. For each of the two periods, a total score of negative life events will be calculated.

#### Motivation to start with the intervention

Three questions will be used to measure the motivation to start the intervention. Two questions will be asked regarding the expected usefulness of the intervention, and one question about the motivation to start with the intervention. Questions will be answered on a five-point scale, and the scores on the items will be summed.

#### Compliance

Two measures of program adherence will be used in this study. During the intervention, program adherence will be measured three times by asking participants whether they read the information on the website and performed the exercises. Furthermore, we will monitor whether the participant calls with the coach. These two measures will be handled separately.

#### Dropout and reasons for dropout

Two types of dropout will be investigated three times during the intervention and at T1, T2, and T3. We will register the participants who stopped using the intervention and the participants who did not complete the questionnaires. We will remind participants to fill in the questionnaires. When they drop out, we will ask them why they do not wish to participate anymore to investigate the reasons for dropout. We will examine the difference in dropout between groups and differences between participants who completed and stopped using the intervention.

#### Medical data

Medical data will be obtained from the Athena/SHM Cohort Study after consent from the participant. The ATHENA Cohort Study is maintained by Stichting HIV Monitoring, which is supported by the Dutch Ministry of Health via the National Institute for Public Health and Environment (RIVM). The data that will be used include the time since HIV diagnosis, medication use, CD4 cell count, and viral load.

#### User satisfaction questionnaire

At T1, we will ask participants about their satisfaction with the self-help program and the coach with a self-designed questionnaire. The questionnaire consists of 31 items to evaluate the intervention, for example, the components of the self-help program and the support from the coach. Open-ended and closed-ended questions will be used in the questionnaire. The questions for participants in the control group will concern the support from the coach (13 questions). The answers to these questions will be used to adjust the self-help program before implementing it.

### Statistical analyses

A two-tailed alpha of .05 will be used for significance testing. All analyses will be based on an intention-to-treat (ITT) analysis, which includes all participants who were randomized to one of the groups at the beginning of the study [47]. Baseline differences between conditions will be investigated with chi-square tests for categorical variables and ANOVAs for continuous variables.

To examine the effect of condition on the depressive symptoms at the post-tests, longitudinal multilevel regression analysis (LMRA) will be conducted [48]. A two-level model will be studied: time at level 1 and participants at level 2. The variable time will have four categories indicating the four time points (T0–T4). The baseline (T0) will be used as reference category. The independent variable is condition, the dependent variable is the score on the PHQ-9 or the CES-D, and several covariates will be included in the model (e.g., sex and age). Multiple imputation for missing data is not necessary in LMRA because it can include participants with missing data on one or more time points [48]. The existence of an effect of HIV treatment center will be investigated exploratory.

Furthermore, Cohen’s d will be calculated to indicate within- and between-group effect sizes [49]. In addition, clinically important differences for individual change in depressive symptoms will be examined. Change scores of 5 or more from the pretest to the post-tests will be considered as clinical important differences for the PHQ-9 [50].

With regard to all secondary outcomes, we will study the pre-treatment versus post-treatment changes by using the analyses described above for the PHQ-9 and the CES-D. To examine moderators of the treatment outcome, LMRA will be conducted. Potential moderators will be entered individually. Interaction effects will be investigated (e.g., time x condition x moderator). A limited number of cross-product interactions will be examined, and we will correct for multiple testing. Furthermore, the QUINT (Qualitative Interaction Trees [51]) method will be used to identify subgroups for which the intervention is most effective, by exploratory investigation of the multiple moderators.

Mediation will be tested by examining the indirect effect of the independent variable (condition) on the dependent variable (depressive symptoms) through the proposed mediators. Mediation analysis will be conducted by using latent difference score models, which are recommended with repeated measurements and multiple mediators [52]. These models focus on intra-individual change and individual differences in that change.

## Discussion

In the proposed study, we will investigate the effectiveness of an online self-help intervention for PLH with depressive symptoms in a randomized controlled trial. Additionally, moderators and mediators of the treatment outcome will be assessed. This study has some strengths and limitations that will be discussed in the following section. First, a strength of this study is that the self-help program is online, so many people can be reached. Only PLH without Internet or an e-mail address cannot participate, but most people in The Netherlands have access to the Internet. Second, participants can work on the program when and where they want and at their own pace. Third, the content of the program is based on prior research and a needs assessment among PLH [14–18]; that is, the program is designed especially for PLH. Fourth, moderators of the treatment outcome will be studied; we will investigate for whom the program is especially beneficial. Investigations of the effect of personal characteristics on the treatment outcome are useful, so patients could receive the treatment that is most likely to decrease their depressive symptoms. Fifth, mediators of the treatment outcome will be assessed; the mechanisms of change of the intervention will be studied. Knowing more about exactly how the invention works is important. Knowing what is effective would allow the adaptation of the treatment to make it more efficient [53]. Sixth, the support participants receive from the coach is also a strength in this study. The coach will motivate participants to continue with the program, and this will probably decrease the dropout. Lastly, most HIV treatment centers in the Netherlands agreed to screen patients for the study. This means that we will have PLH from across the country participating in the study.

Furthermore, some limitations are present in the study. Patients with severe depressive symptoms and/or suicidal ideation will be excluded from the study. We argue that these patients should be closely monitored by a therapist. We expect that these patients would benefit from the self-help program, as has also been found in previous research [54], but more intense treatment is desirable for these patients. In addition, the follow-up period of participants in the control group is only 3 months (they may start with the intervention after the 3-month follow-up), while this period is 6 months for the participants in the intervention group. However, we argue that it is not ethical to let people in the control group wait for approximately 8 months before providing the intervention (8 weeks plus 6 months of follow-up). Therefore, the intervention will be offered to the control group after the second post-test. The last limitation concerns the screening of participants for depressive symptoms. We will use a questionnaire with nine items to measure depressive symptoms in PLH. One could argue that a diagnostic interview will screen depressive symptoms more extensively and allow the identification of a possible depressive disorder. However, this approach would be time consuming, and the PHQ-9 is a reliable and valid measure of severity of depressive symptoms [37]. Furthermore, the program is designed for people with mild to moderate depressive symptoms, so a diagnosis of depression is not an inclusion criterion for participation in the study.

To conclude, in the proposed study, we will evaluate an online self-help program for PLH with depressive symptoms. Since depressive symptoms exist frequently in PLH, low-cost and low-stigmatizing interventions are needed for these individuals. The proposed study offers an online self-help program that complies with this request. If the self-help program proves to be effective in the proposed study, implementation of the website will be done in cooperation with the Dutch patient organization (HIV Vereniging Nederland) and HIV treatment centers. Furthermore, the program may be implemented in other countries as well.

### Trial status

Patient recruitment is completed.

## Additional file

Additional file 1: SPIRIT Checklist. (DOC 120 kb)

## Abbreviations

BADS: Behavioral Activation for Depression Scale
BERQ: Behavioral Emotion Regulation Questionnaire
CBT: cognitive behavioral therapy
CCI: Crandell Cognitions Inventory
CD4: cluster of differentiation 4
CERQ: Cognitive Emotion Regulation Questionnaire
CES-D: Center of Epidemiologic Studies Depression Scale
ERQ: Emotion Regulation Questionnaire
GAD-7: Generalized Anxiety Disorder 7
HAART: highly active antiretroviral therapy
HADS: Hospital Anxiety and Depression Scale
ICC: intracluster correlation coefficient
ITT: intent-to-treat
LMRA: longitudinal multilevel regression analysis
LUMC: Leiden University Medical Center
PASS: Power Analysis and Sample Size Software
PHQ-2: Patient Health Questionnaire 2
PHQ-4: Patient Health Questionnaire 4
PHQ-9: Patient Health Questionnaire 9
PLH: people living with HIV
QUINT: Qualitative Interaction Trees

## References

[1] Cruess DG, Evans DL, Repetto MJ, Gettes D, Douglas SD, Petitto JM (2003). Prevalence, diagnosis, and pharmacological treatment of mood disorders in HIV disease. Biol Psychiatry.

[2] Sherr L, Clucas C, Harding R, Sibley E, Catalan J (2011). HIV and depression--a systematic review of interventions. Psychol Health Med.

[3] Bravo P, Edwards A, Rollnick S, Elwyn G (2010). Tough decisions faced by people living with HIV: a literature review of psychosocial problems. AIDS Rev.

[4] Gonzalez JS, Batchelder AW, Psaros C, Safren SA (2011). Depression and HIV/AIDS treatment nonadherence: a review and meta-analysis. J Acquir Immune Defic Syndr.

[5] Brown JL, Vanable PA (2008). Cognitive-behavioral stress management interventions for persons living with HIV: a review and critique of the literature. Ann Behav Med.

[6] Carrico AW, Antoni MH (2008). Effects of psychological interventions on neuroendocrine hormone regulation and immune status in HIV-positive persons: a review of randomized controlled trials. Psychosom Med.

[7] Crepaz N, Passin WF, Herbst JH, Rama SM, Malow RM, Purcell DW (2008). Meta-analysis of cognitive-behavioral interventions on HIV-positive persons’ mental health and immune functioning. Health Psychol.

[8] Scott-Sheldon LAJ, Kalichman SC, Carey MP, Fielder RL (2008). Stress management interventions for HIV+ adults: a meta-analysis of Randomized controlled trials, 1989 to 2006. Health Psychol.

[9] Spies G, Asmal L, Seedat S (2013). Cognitive-behavioural interventions for mood and anxiety disorders in HIV: a systematic review. J Affect Disord.

[10] Swendeman D, Ingram BL, Rotheram-Borus MJ (2009). Common elements in self-management of HIV and other chronic illnesses: an integrative framework. AIDS Care.

[11] Cuijpers P, Donker T, van Straten A, Li J, Andersson G (2010). Is guided self-help as effective as face-to-face psychotherapy for depression and anxiety disorders? A systematic review and meta-analysis of comparative outcome studies. Psychol Med.

[12] Gellatly J, Bower P, Hennessy S, Richards D, Gilbody S, Lovell K (2007). What makes self-help interventions effective in the management of depressive symptoms? Meta-analysis and meta-regression. Psychol Med.

[13] Williams C, Martinez R (2008). Increasing Access to CBT: stepped care and cbt self-help models in practice. Behav Cogn Psychother.

[14] Garnefski N, Kraaij V, Schroevers MJ (2010). Leven met HIV: zelfhulpboek voor het omgaan met HIV [Living with HIV: self-help book for coping with HIV].

[15] Kraaij V, Garnefski N, Schroevers MJ, van der Veek SM, Witlox R, Maes S (2008). Cognitive coping, goal self-efficacy and personal growth in HIV-infected men who have sex with men. Patient Educ Couns.

[16] Kraaij V, van der Veek SM, Garnefski N, Schroevers M, Witlox R, Maes S (2008). Coping, goal adjustment, and psychological well-being in HIV-infected men who have sex with men. AIDS Patient Care STDS.

[17] van der Veek SM, Kraaij V, Van Koppen W, Garnefski N, Joekes K (2007). Goal disturbance, cognitive coping and psychological distress in HIV-infected persons. J Health Psychol.

[18] Kraaij V, van Emmerik A, Garnefski N, Schroevers MJ, Lo-Fo-Wong D, van Empelen P (2010). Effects of a cognitive behavioral self-help program and a computerized structured writing intervention on depressed mood for HIV-infected people: a pilot randomized controlled trial. Patient Educ Couns.

[19] van Beugen S, Ferwerda M, Hoeve D, Rovers MM, Spillekom-van Koulil S, van Middendorp H (2014). Internet-based cognitive behavioral therapy for patients with chronic somatic conditions: a meta-analytic review. J Med Internet Res.

[20] Clarke G, Debar L, Lynch F, Powell J, Gale J, O’Connor E (2005). A Randomized effectiveness trial of brief cognitive-behavioral therapy for depressed adolescents receiving antidepressant medication. J Am Acad Child Adolesc Psychiatry.

[21] Garnefski N, Kraaij V, Benoist M, Bout Z, Karels E, Smit A (2013). Effect of a cognitive behavioral self-help intervention on depression, anxiety, and coping self-efficacy in people with rheumatic disease. Arthritis Care Res (Hoboken).

[22] Kroenke K, Spitzer RL, Williams JBW (2001). The PHQ-9 - Validity of a brief depression severity measure. J Gen Intern Med.

[23] NCSS and PASS (number cruncher statistical systems), www.NCSS.com. Accessed 5 Mar 2014.

[24] Kroenke K, Spitzer RL, Williams JBW (2003). The Patient Health Questionnaire-2 - validity of a two-item depression screener. Med Care.

[25] Zigmond AS, Snaith RP (1983). The Hospital Anxiety and Depression Scale. Acta Psychiatr Scand.

[26] Radloff LS (1977). The CES-D scale: a self-report depression scale for research in the general population. Appl Psychol Meas.

[27] Kanter JW, Mulick PS, Busch AM, Berlin KS, Martell CR (2007). The Behavioral Activation for Depression Scale (BADS): Psychometric Properties and Factor Structure. J Psychopathol Behav Assess.

[28] Gross JJ, John OP (2003). Individual differences in two emotion regulation processes: Implications for affect, relationships, and well-being. J Pers Soc Psychol.

[29] Garnefski N, Kraaij V, Spinhoven P (2001). Negative life events, cognitive emotion regulation and emotional problems. Pers Indiv Differ.

[30] Crandell CJ, Chambless DL (1986). The validation of an inventory for measuring depressive thoughts - the Crandell Cognitions Inventory. Behav Res Ther.

[31] Wrosch C, Scheier MF, Miller GE, Schulz R, Carver CS (2003). Adaptive self-regulation of unattainable goals: goal disengagement, goal reengagement, and subjective well-being. Pers Soc Psychol Bull.

[32] Spitzer RL, Kroenke K, Williams JBW, Lowe B (2006). A brief measure for assessing generalized anxiety disorder - the GAD-7. Arch Intern Med.

[33] Life Events Scale, www.cerq.leidenuniv.nl. Accessed 2 Sept 2014.

[34] Donker T, Batterham PJ, Warmerdam L, Bennett K, Bennett A, Cuijpers P (2013). Predictors and moderators of response to Internet-delivered interpersonal psychotherapy and cognitive behavior therapy for depression. J Affect Disord.

[35] Warmerdam L, Van Straten A, Twisk J, Cuijpers P (2013). Predicting outcome of Internet-based treatment for depressive symptoms. Psychother Res.

[36] Kroenke K, Spitzer RL, Williams JBW, Löwe B (2009). An ultra-brief screening scale for anxiety and depression: the PHQ–4. Psychosomatics.

[37] Kroenke K, Spitzer RL, Williams JB, Lowe B (2010). The Patient Health Questionnaire Somatic, Anxiety, and Depressive Symptom Scales: a systematic review. Gen Hosp Psychiatry.

[38] Relf MV, Eisbach S, Okine KN, Ward T (2013). Evidence-based clinical practice guidelines for managing depression in persons living with HIV. J Assoc Nurses AIDS Care.

[39] Thombs BD, Hudson M, Schieir O, Taillefer SS, Baron M, Canadian Scleroderma Research Group (2008). Reliability and validity of the Center for Epidemiologic Studies Depression Scale in patients with systemic sclerosis. Arthritis Rheum.

[40] Tsai AC (2014). Reliability and validity of depression assessment among persons with hiv in sub-Saharan Africa: systematic review and meta-analysis. J Acquir Immune Defic Syndr.

[41] Simoni JM, Safren SA, Manhart LE, Lyda K, Grossman CI, Rao D (2011). Challenges in addressing depression in HIV research: assessment, cultural context, and methods. AIDS Behav.

[42] Raes F, Hoes D, Van Gucht D, Kanter JW, Hermans D (2010). The Dutch version of the Behavioral Activation for Depression Scale (BADS): psychometric properties and factor structure. J Behav Ther Exp Psychiatry.

[43] Garnefski N, Kraaij V (2007). The Cognitive Emotion Regulation Questionnaire - psychometric features and prospective relationships with depression and anxiety in adults. Eur J Psychol Assess.

[44] Glass CR, Arnkoff DB (1997). Questionnaire methods of cognitive self-statement assessment. J Consult Clin Psychol.

[45] Schwarzer R, Jerusalem M, Weinman J, Wright S, Windsor JM (1995). Generalized Self-Efficacy Scale. Measures in health psychology: a user’s portfolio. Causal and control beliefs.

[46] Spinhoven PH, Ormel J, Sloekers PPA, Kempen GIJM, Speckens AEM, Hemert AMV (1997). A validation study of the Hospital Anxiety and Depression Scale (HADS) in different groups of Dutch subjects. Psychol Med.

[47] Hollis S, Campbell F (1999). What is meant by intention to treat analysis? Survey of published randomised controlled trials. Br Med J.

[48] Stoel RD, van Den Wittenboer G, Hox J (2003). Analyzing longitudinal data using multilevel regression and latent growth curve analysis. Metodologia de las Ciencias del Comportamiento.

[49] Cohen J (1992). A power primer. Psychol Bull.

[50] Lowe B, Unutzer J, Callahan CM, Perkins AJ, Kroenke K (2004). Monitoring depression treatment outcomes with the patient health questionnaire-9. Med Care.

[51] Dusseldorp E, Doove L, Van Mechelen I: Quint: An R package for identification of subgroups of clients who differ in which treatment alternative is best for them. Behav Res Methods 2015. (in press). 10.3758/s13428-015-0594-z PMC4891398 26092391

[52] Selig JP, Preacher KJ (2009). Mediation models for longitudinal data in developmental research. Res Hum Dev.

[53] Kazdin AE (2007). Mediators and mechanisms of change in psychotherapy research. Annu Rev Clin Psychol.

[54] van Straten A, Cuijpers P, Smits N (2008). Effectiveness of a web-based self-help intervention for symptoms of depression, anxiety, and stress: randomized controlled trial. J Med Internet Res.

